# Full of Beans? Early Soy Exposure Associated with Less Feminine Play in Girls

**DOI:** 10.1289/ehp.119-a525b

**Published:** 2011-12-01

**Authors:** Tanya Tillett

**Affiliations:** Tanya Tillett, MA, of Durham, NC, is a staff writer/editor for *EHP*. She has been on the *EHP* staff since 2000 and has represented the journal at national and international conferences.

Animal studies have shown that sexually dimorphic behavior can be influenced by soy isoflavones—compounds with a structural and functional similarity to estrogen—but there is a lack of information regarding the effects of soy isoflavone exposure on postnatal child development. A new study reports an association between early soy feeding and less female-typical play behavior in young girls **[*EHP* 119(12):1811–1816; Adgent et al.]**.

The authors analyzed gender role-play behaviors among 3,664 boys and 3,412 girls enrolled in the United Kingdom Avon Longitudinal Study of Parents and Children. They used feeding data from questionnaires completed by mothers at 1, 6, 15, and 24 months post-partum to divide children into four categories: “primarily breast” (breastfed for at least 6 months), “early formula” (introduced to nonsoy milk or infant formula at or before 4 months old, with sustained use at 6 months of age), “early soy” (introduced to soy milk or soy formula at or before 4 months old, with sustained use at 6 months of age), and “late soy” (introduced to soy milk or soy formula anytime between 5 and 15 months of age). The Preschool Activities Inventory (PSAI), which measures how often a child plays with certain toys, engages in certain activities, and displays certain characteristics over a month’s time based on “masculine” or “feminine” classification, was used to assess gender-role play behavior at 30, 42, and 57 months of age.

**Figure:**
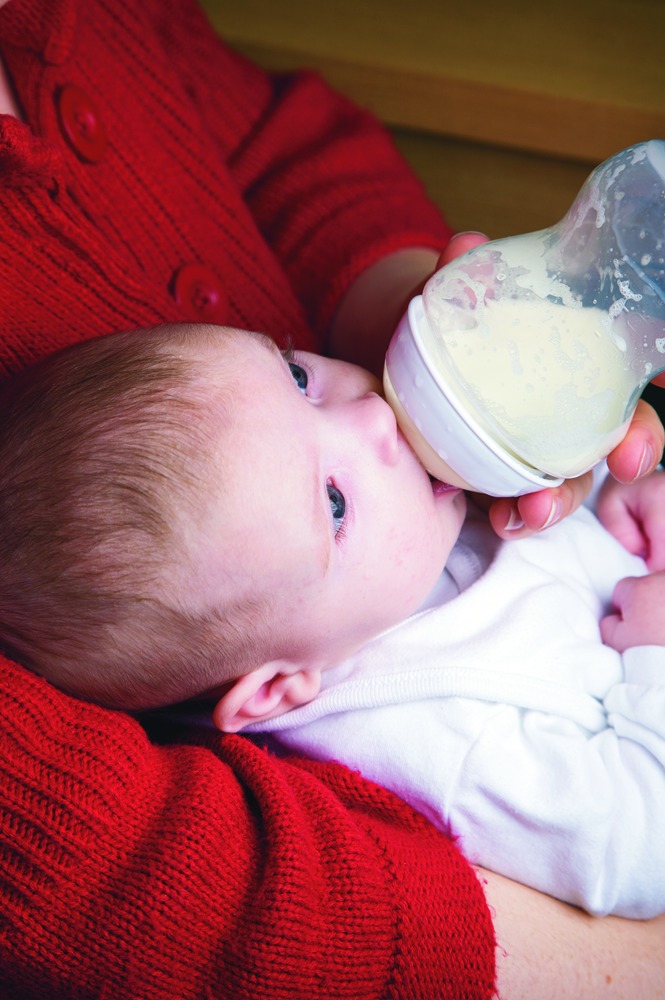
Early exposure to soy infant formula was associated with less female-typical play in girls at age 42 months. © domturner/Shutterstock.com

Focusing on outcomes at age 42 months to correspond with other, similar studies, the investigators found higher PSAI scores (indicating less typically feminine play) among “early soy” girls compared with “early formula” girls, but scores remained in the normal range for female play behavior. They saw no significant difference in girls’ behavior when breastfeeding and early formula feeding were compared. They also observed marginally higher PSAI scores among “early soy” boys compared with other boys, and noted the lowest PSAI scores among boys who were primarily breastfed. However, no significant difference was observed between “early soy” and “early formula” boys. In both sexes, PSAI scores were higher if an older brother were present in the home and lower if an older sister were present. Maternal prenatal smoking also was associated with higher PSAI scores in both sexes, and higher maternal education was associated with lower scores in boys and higher scores in girls.

The authors acknowledge that soy users in the study were not exclusively fed soy formula in all instances, nor could they assess a dose–response relationship between soy feeding and PSAI score. These preliminary data suggest a subtle reduction in female-typical play behaviors at age 42 months in girls who were fed soy formula or soy milk early in life—an association that weakened by age 57 months. Replication of these findings in cohorts with more prevalent soy use and improved exposure assessment is needed.

